# Mass Spectrometry Imaging of Drug Related Crystal-Like Structures in Formalin-Fixed Frozen and Paraffin-Embedded Rabbit Kidney Tissue Sections

**DOI:** 10.1007/s13361-015-1254-3

**Published:** 2015-09-18

**Authors:** Anne L. Bruinen, Cateau van Oevelen, Gert B. Eijkel, Marjolein Van Heerden, Filip Cuyckens, Ron M. A. Heeren

**Affiliations:** M4I, Maastricht University, Universiteitssingel 50, 6229 ER Maastricht, The Netherlands; Preclinical Development and Safety, Janssen Research and Development, Turnhoutseweg 30, 2340 Beerse, Belgium

**Keywords:** Imaging mass spectrometry, SIMS, MALDI, DESI, Multimodal, Metabolites

## Abstract

A multimodal mass spectrometry imaging (MSI) based approach was used to characterize the molecular content of crystal-like structures in a frozen and paraffin embedded piece of a formalin-fixed rabbit kidney. Matrix assisted laser desorption/ionization time-of-flight (MALDI-TOF) imaging and desorption electrospray ionization (DESI) mass spectrometry imaging were combined to analyze the frozen and paraffin embedded sample without further preparation steps to remove the paraffin. The investigated rabbit kidney was part of a study on a drug compound in development, in which severe renal toxicity was observed in dosed rabbits. Histological examination of the kidney showed tubular degeneration with precipitation of crystal-like structures in the cortex, which were assumed to cause the renal toxicity. The MS imaging approach was used to find out whether the crystal-like structures were composed of the drug compound, metabolites, or an endogenous compound as a reaction to the drug administration. The generated MALDI-MSI data were analyzed using principal component analysis. In combination with the MS/MS results, this way of data processing demonstrates that the crystal structures were mainly composed of metabolites and relatively little parent drug.

**Graphical Abstract:**
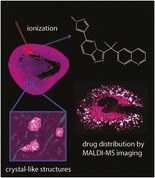
ᅟ

ᅟ

## Introduction

Mass spectrometry imaging (MSI) is an increasingly popular technique providing insight into the molecular distribution inside thin tissue samples [1]. In contrast, traditional histopathologic investigation can only visualize structural abnormalities.

Matrix assisted laser desorption ionization mass spectrometry imaging (MALDI-MSI) is such a technique that is able to record molecular images of tissue sections. Ions are generated from the sample surface by irradiating it with a UV laser [2, 3]. The laser moves over the sample in an array of spots, and a spectrum is acquired from the ionized species on every position. The information obtained from the whole sample is reconstructed into a 2D image. The same principle is used in desorption electrospray ionization (DESI), where the ionization is attributed to charged droplets that hit the sample surface spot by spot. After dissolution of the ionized analytes, the charged droplets travel into a mass spectrometer to be analyzed [1, 4–6]. The power of MSI over light microscopy is the ability to identify compounds in combination with structural abnormalities in tissues.

Pathologists commonly embed formalin-fixed tissues in paraffin. Formalin fixation results in the cross-linking of proteins and paraffin embedding preserves the tissue morphology, makes it easier to cut sections which are large or fragile, and creates the possibility of long-term storage. Formalin-fixed and paraffin-embedded (FFPE) samples are ideal for microscopic investigation after contrast staining.

However, protein cross-linking caused by formalin fixation is not favorable for MSI, since mass spectrometry imaging is based on the mapping of molecules, including these cross-linked proteins. Besides, formalin fixation causes the formation of sodium adducts and paraffin causes severe ion suppression [7]. In general, the risk of delocalization of compounds from the tissue into the embedding material is high. Several wash steps are usually required to avoid ion suppression and the formation of sodium adducts, with an associated risk of delocalization and washing away analytes causing a lower concentration as a result.

It is essential not to alter the morphology and composition of the biological tissue prior to analysis of biological tissue samples using mass spectrometry imaging [7, 8]. This ensures that the best sensitivity, accuracy, and reproducibility can be obtained [7, 9]. It appears that freezing of a tissue by free-floating in liquid nitrogen after sampling from an organism gives the best preservation. Sometimes dry-ice chilled isopentane or hexane is used for larger samples to prevent fracturing [9–11].

In this study, we show the combination of MALDI and DESI mass spectrometry imaging to detect, map, and identify a drug molecule and its major metabolites in a formalin-fixed (FF) frozen kidney sample with a formalin-fixed paraffin-embedded (FFPE) kidney sample. Principal component analysis (PCA) of the MALDI-MSI data was used as a tool to give insight into the contribution of different molecules to the renal crystal structures.

**Figure 1:**
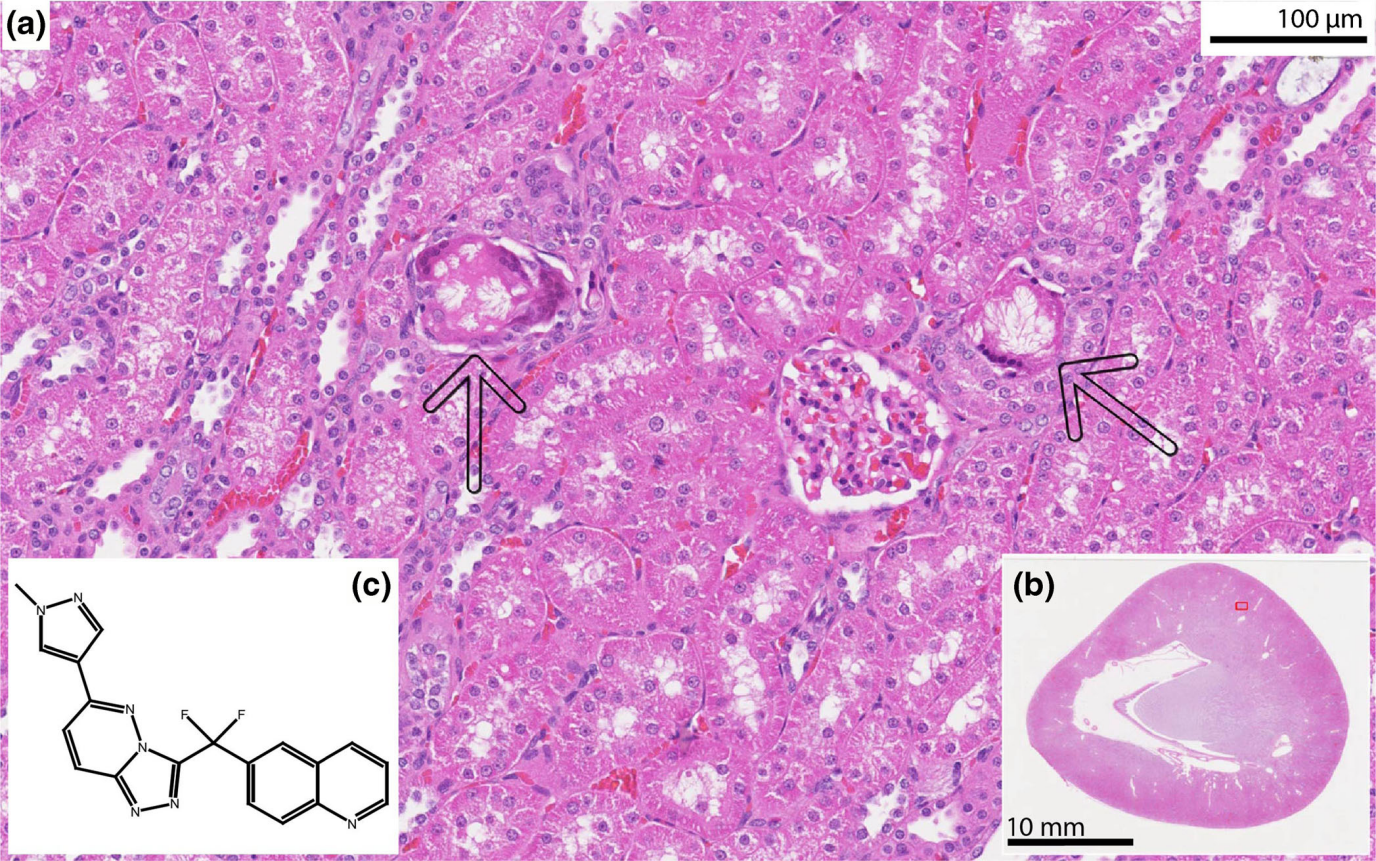
**(a)** Histology of a hematoxylin and eosin stained section from a formalin-fixed paraffin-embedded kidney of a dosed rabbit. The arrows point out the cortical intratubular crystalline material. The insert **(b)** is an overview of the kidney. **(c)** Chemical structure of the drug compound in the pipeline

A c-Met tyrosine kinase inhibitor in development was administered by oral gavage to male rabbits for 1 month as part of toxologic research and the drug’s potential as an anti-cancer drug (Figure 1c) [12]. Renal toxicity was observed in the dosed rabbits in a similar way as described in reference [13]. The kidney weights were increased and kidneys showed pale discoloration or discolored foci at necropsy. Histopathological examination of kidneys showed degenerative changes composed of dilatation of collecting ducts and cortical tubules, multifocal vacuolization, and tubular hypertrophy. Within the lumen of these tubules, birefringent (in polarized light) granular or crystalline material was observed (Figure 1a, b). To test whether the drug compound or one or several of its metabolites caused this structure formation, MSI was considered to be a suitable technique to reveal their molecular content [14].

Less interference from either paraffin itself or cross-linked species was expected in paraffin-embedded tissue from this study on a low molecular drug compound than for a study on high mass molecules. By evaluating the MALDI-MSI and DESI-MSI feasibility and performance on tissue slides prepared and stored within formalin, but also embedded in paraffin, as is typically done in toxicity studies, would enable a combined analysis of the same tissue samples with light microscopy and MSI [15, 16].

## Experimental

### Tissue Sectioning

Half a formalin-fixed kidney from a 1-month repeated dose oral toxicity study in male rabbits with the drug compound in development was obtained from Janssen R&D (Beerse, Belgium). The specimen was frozen at –20°C overnight. Sections of 20 and 30 μm thickness were cut using a cryo-microtome (HM525; MICROM Walldorf, Germany and CM3050 S; Leica, Wetzlar, Germany, respectively) and thaw mounted on Superfrost microscope glass slides (Thermo Scientific, Waltham, Massachusetts USA).

The other half of the formalin-fixed kidney described above was provided embedded in paraffin (FFPE) by Janssen R&D (Beerse, Belgium); 8 and 10 μm thick sections were cut using a cryo-microtome (CM3050 S; Leica) and mounted on Superfrost microscope glass slides (Thermo Scientific).

### MALDI Imaging

The slides with frozen tissue sections were dried in a vacuum desiccator for at least 15 min before matrix deposition. A solution of 20 mg/mL of 2,5-dihydroxybenzoic acid (DHB) in 1:1 acetonitrile (ACN):0.1% 2,2,2-trifluoroethanoic acid (TFA) in H_2_O was prepared and deposited in 40 spray cycles on all glass slides using a SunCollect MALDI-spotter (SunChrom, Friedrichdorf Germany). In the first cycle, a flow rate of 5 μL/min was used, in the second 10 μL/min, in the third 15 μL/min, and 30 μL/min for the remaining cycles.

The 20 μm frozen kidney and 10 μm FFPE tissue samples were analyzed on a SYNAPT G1 HDMS Q-ToF instrument with a MALDI ionization source equipped with a 200 Hz Nd:YAG laser (355 nm) (Waters Corporation, Milford, MA, USA). The system was running in positive ionization mode with 10,000 mass resolution at *m/z 556* in V-ToF mode and analyzing *m/z* 50–500. Images were acquired with a sample stage step size of 150 μm.

The raw data were converted with a *m/z* bin size of 0.1 to a suitable format for BioMap (Novartis, Basel, Switzerland). The ion images and their overlays with the optical scan images of the tissue sections were generated with the same software.

Multivariate analysis was performed with the in-house written ChemomeTricks toolbox for MATLAB (ver. 7.0, The MathWorks) to determine the composition of the crystal-like structures. Principal component analysis (PCA) was employed to find sets of correlated mass channels that represent the maximum amount of variance.

Prior to the MS/MS imaging analysis, 1 μL of 500 μM of the drug compound in 1:1 ACN:H_2_O mixed 1:1 (v/v) with the DHB matrix solution was dry-spotted on a MALDI target plate. Product ion spectra were generated through the fragmentation of a precursor ion at *m/z* 378 (corresponding to the mass of the protonated drug molecule) with a collision energy of 50 eV in the trap collision cell of the Synapt instrument. The same 500 μM solution in 1:1 ACN:H_2_O was deposited as a spot next to the formalin-fixed frozen kidney tissue section before matrix deposition. An area including this spotted standard was selected to perform an MS/MS imaging experiment with fragmentation of *m/z* 380, related to the major metabolite of the drug compound. BioMap software was also used here to generate overlay images and correlate the fragments observed in the tissue with the ones in the spotted standard.

### DESI Imaging

A 30 μm frozen kidney tissue section and an 8 μm FFPE tissue section were analyzed on a Q Exactive Hybrid Quadrupole-Orbitrap mass spectrometer (Thermo Scientific, Bremen, Germany), operating in positive ionization mode and detecting the *m/z* 350–500 mass range. A mass resolution of 70,000 (at *m/z* 200) was chosen for the frozen tissue section, and a resolution of 140,000 (at *m/z* 200) for the paraffin embedded section to obtain more selectivity.

An Acquity pump (Waters, Milford, MA, USA) set at 800 μL/min flow rate was used to generate a DESI spray. A homemade flow splitter was used to reduce the flow rate to 2.5 μL/min at the DESI emitter, which was a 50 μm fused silica capillary (Prosolia, Indianapolis, IN, USA) on a 3.5 kV high voltage with 7.6 bar nitrogen gas pressure. The distance between the emitter and the sample was ~3 mm and the emitter and the MS inlet were separated by ~10 mm. The distance between the MS inlet and the sample was kept as small as possible. Chloroform, acetonitrile and formic acid (1:1:0.1v%) were used as spraying solvent.

DESI images were generated using a surface scan rate of 100 μm/s (1 s/scan at 70,000 resolution and 2 microscans) for the formalin-fixed frozen tissue section and 130 μm/s (0.77 s/scan at 140,000 resolution and 1 microscan) for the formalin-fixed paraffin-embedded section. This resulted in tissue section images with a 100 μm pixel size. The DESI images were mapped using the BioMap software (Novartis) as described above.

## Results and Discussion

MALDI and DESI imaging experiments were performed on a frozen and a paraffin-embedded part of a formalin-fixed kidney to explore the possibility of analysis on typically prepared samples for histology. We show the potential of this approach with the research into crystal-like structures found in rabbit kidneys and whether they could be directly linked to the drug compound or its metabolites that was administered to the animals.

**Figure 2:**
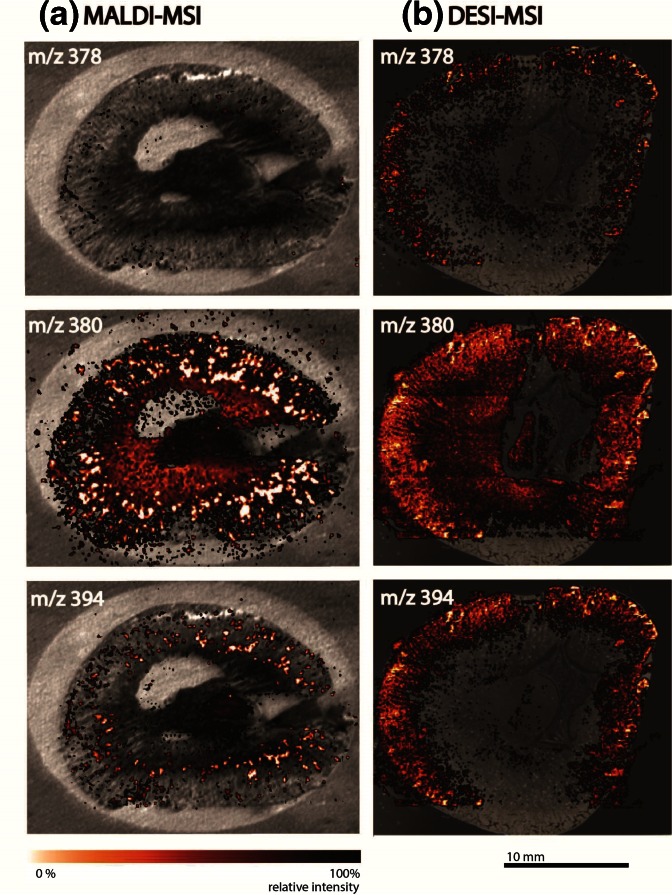
MALDI and DESI image overlays representing the spatial distribution of the drug compound (*m/z* 378) and its most abundant metabolites (*m/z* 380 and 394) in a tissue section of a formalin-fixed frozen rabbit kidney

The spatial distribution of molecular compounds in the FF frozen tissue section of a kidney obtained from a dosed rabbit was directly visualized using BioMap software. The drug compound has a ^12^C monoisotopic mass of 377 Da and was expected to show up in the protonated form at *m/z* 378. The *m/z* 380 peak corresponds to the protonated molecule of the metabolite originating from demethylation of the tertiary amine on the imidazole ring resulting in a secondary amine with a proton instead of a methyl group [(–15 + 1)= –14 Da] and oxidation of the quinoline ring (+16 Da) from the parent drug. The peak at *m/z* 394 was linked to a protonated molecule of the metabolite. These masses were plotted as overlays of the molecular distribution with the optical image of the tissue section in Figure 2 for both MALDI **(a)** and DESI **(b)** imaging experiments.

At first sight, the pixels with the highest intensities for the *m/z* values corresponding to the drug compound and two metabolites seem to be present on the same location and in the shape of dots (Figure 2). They appear mainly in the cortex where the crystals were found during histopathologic investigation (Figure 1) [12].

**Figure 3:**
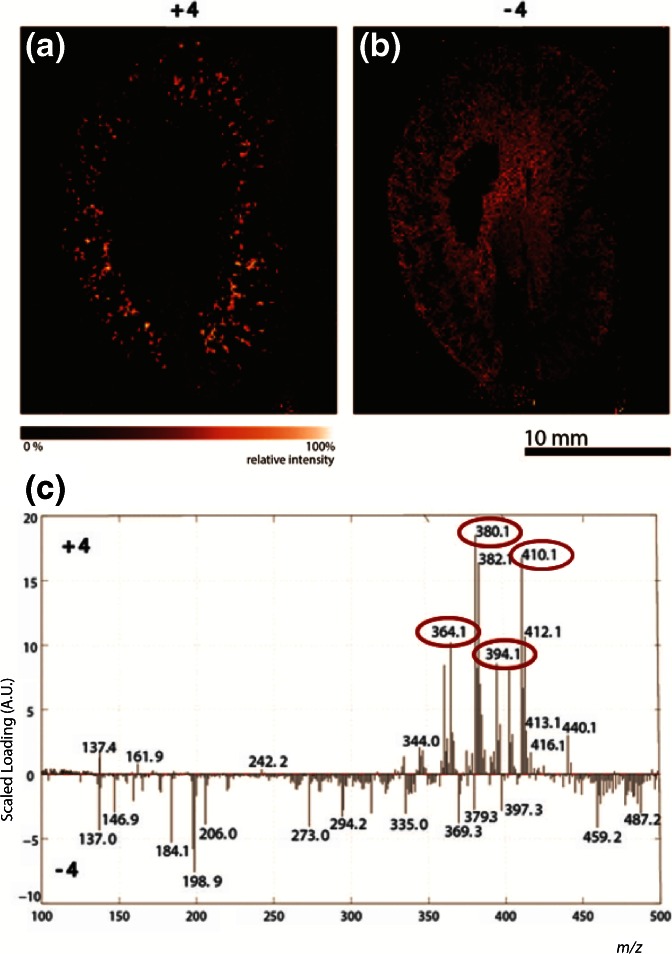
Images describing the positive **(a)** and negative **(b)** part of principal component 4 of a frozen formalin-fixed kidney tissue section and their corresponding loading plots **(c)**. The most abundant metabolite (*m/z* 380) and also known metabolites with *m/z* values of 394 and 364 are circled

In order to identify the compounds that jointly constitute the crystal-like structures in the cortex of the kidney, principal component analysis (PCA) was performed on the MALDI-MSI data. PCA is a multivariate type of analysis that is widely used to reduce the size of mass spectrometry imaging data sets by describing variables with more relevant principal components. By using this approach, correlation between variables can be revealed, as well as correlations between samples [17, 18]. Figure 3c represents the loading plot of principal component number 4. The selection of this principal component was based on the presence of a high abundant *m/z* 380 peak, which corresponds to the demethylated and oxidized metabolite of the drug compound. Besides *m/z* 380, also *m/z* 364 (demethylation), *m/z* 394 (oxidation), and *m/z* 410 (double oxidation) contribute to the positive part of PC 4 and are circled in red. By means of these plots, distribution images were reconstructed as shown in (a) and (b), were a is the positive part of the plot representing the crystal pattern, and b the negative part, respectively.

The PCA on the MALDI-MSI data confirms the observation that the metabolites mainly precipitate in the cortex of the kidney. The positive part of the principal component appears again as a dotted pattern in the cortex of the kidney tissue (Figure 3).

**Figure 4:**
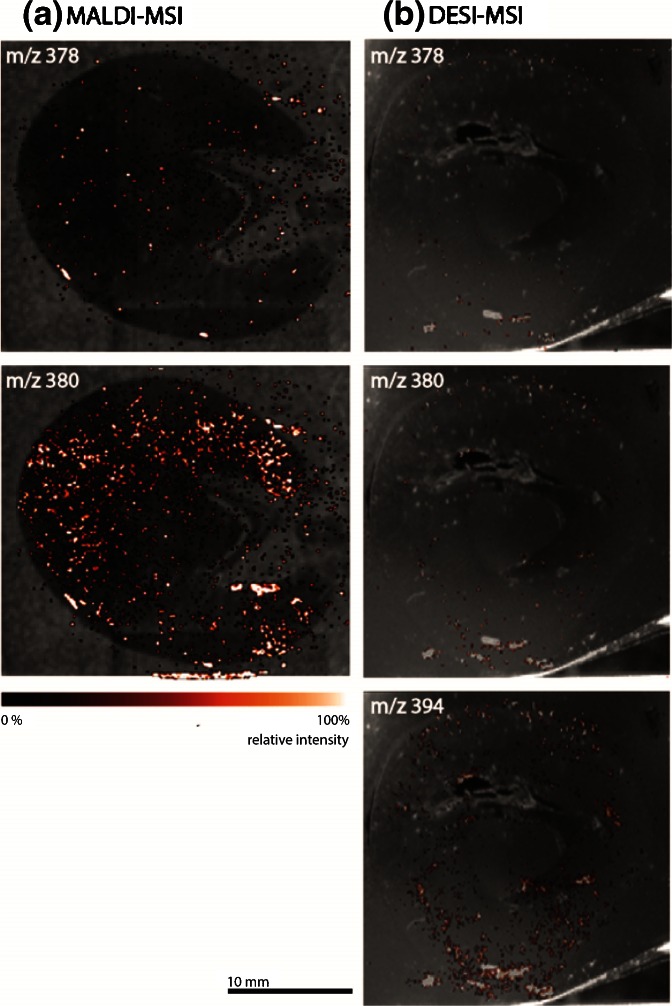
MALDI and DESI image overlays representing the spatial distribution of the drug compound (*m/z* 378) and its most abundant metabolites (*m/z* 380 and 394) in a tissue section of a formalin-fixed and paraffin-embedded rabbit kidney

The same experiment as described above was performed using the FFPE sample. The overlayed ion images of *m/z* 378, *m/z* 380 and *m/z* 394 obtained by MALDI **(a)** and DESI **(b)** MSI are represented in Figure 4. The peaks at *m/z* 378 and 380 appear in a similar pattern as in the frozen tissue sample, though ion intensity for the parent drug (*m/z* 378) is much lower. Also, the metabolite at *m/z* 394 could not be detected with MALDI-MSI under the current conditions. These intensity losses and differences in intensities of the metabolites can be due to washing out the compounds of interest during FFPE sample preparation, ion suppression during the ionisation process, or both.

**Figure 5:**
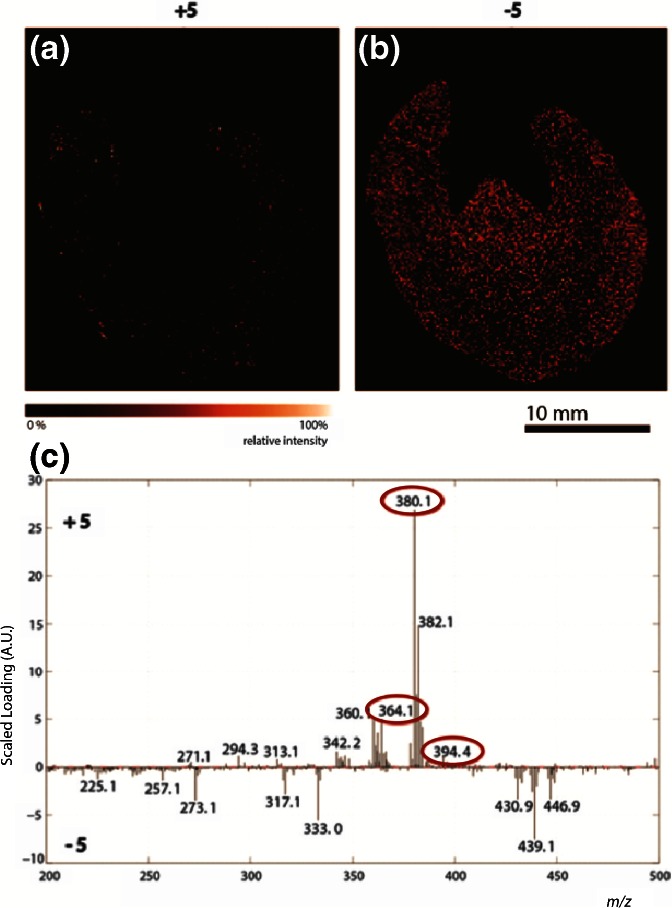
MALDI images describing the positive **(a)** and negative **(b)** part of principal component 5 of a FFPE kidney tissue section and their corresponding loading plots **(c)**. The most abundant metabolite (*m/z* 380) and also known metabolites with *m/z* values of 394 and 364 are circled

PCA was also performed on this MALDI-MSI data set, and for this sample, the fifth PC was selected. The loading plots (c) and correlating reconstructed images of the positive and negative side of PC 5 (a) and (b) are shown in Figure 5. The *m/z* 364 (demethylation) and *m/z* 394 (oxidation) peaks are present in the positive part of PC 5 as well.

**Figure 6:**
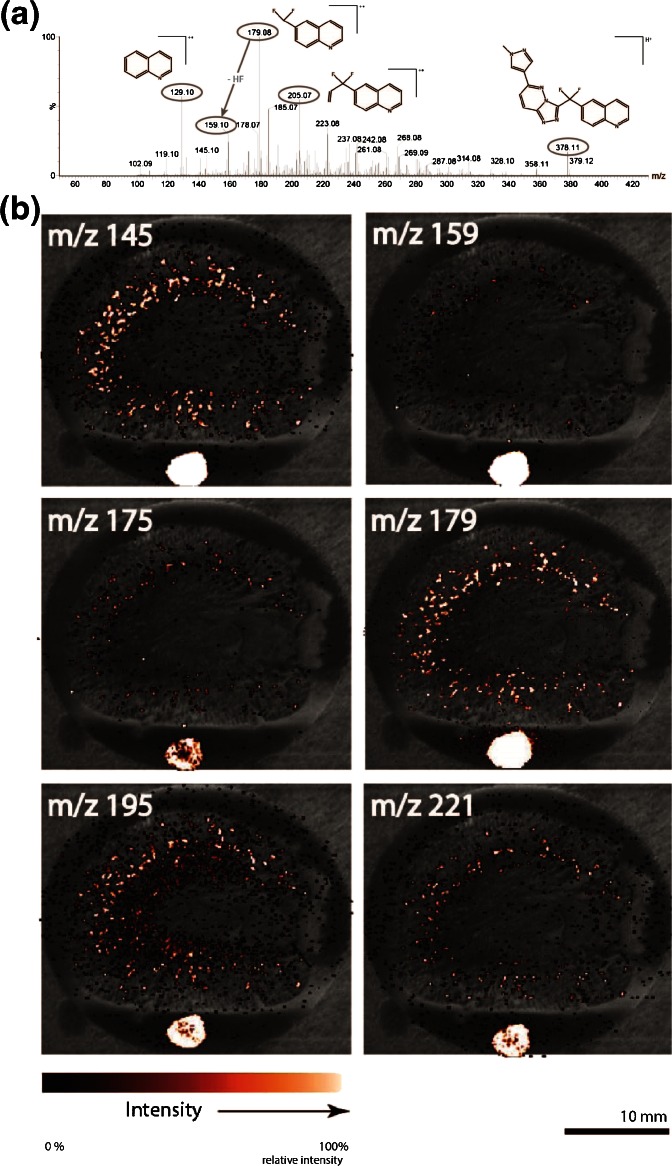
**(a)** MALDI-MS/MS product ion spectrum obtained from the dry spotted drug standard. The main fragments are circled and accompanied by their tentative structures. **(b)** MALDI-MS/MS image of the selected *m/z* 380 peak overlays representing the spatial distribution of product ions present in a tissue section of a formalin-fixed frozen rabbit kidney. The peaks at *m/z* 159 and 179 are linked to fragments found in the drug standard, whereas *m/z* 145 (129+16), 175 (159+16), 195 (179+16), and 221 (205+16) correspond to the oxidized state of fragments found in the drug standard

A MALDI-MS/MS product ion experiment was performed to correlate the precursor masses found at the location of the intratubular cortical crystal-like structures with the standard of the drug compound. Figure 6a shows the product ion spectrum obtained for the drug standard by MALDI-QTOF analysis. The major product ions are annotated with their tentative structure. A MALDI-MS/MS product ion imaging experiment with *m/z* 380 as a precursor was performed to identify the masses found in the crystal-like structures. Figure 6b shows the spatial distribution of the most abundant peaks that appear in both the spotted standard and the tissue area. All these fragments could be correlated to the known fragments originating from the drug compound (*m/z* 159 and 179), or to fragments shifted by 16 Da because of oxygen addition (*m/z* 145, 175, 195, and 221).

As mentioned before, the crystal-like structures were found in the cortex of the rabbit kidneys. It became clear from the overlays in Figure 6b that the molecular fragments from the drug were mostly present in that area. Besides, the same dotted pattern as the crystal-like structures was found in the optical images and previous ion distribution images. The presence of the +16 Da shifted (oxidized) fragments at *m/z* 145, 175, 195, and 221 indicates that based on the tentative product ion structures shown in Figure 6a, the oxidation on the metabolites constituting the crystal-like structures should be located at the quinoline moiety of the drug compound.

## Conclusions

Complementary MALDI and DESI mass spectrometry imaging measurements of the formalin-fixed and frozen kidney samples showed that the crystal-like structures observed in the kidney of dosed rabbits are predominantly composed of metabolites originating from demethylation and/or oxidation and only minor concentrations of the drug compound itself.

The identification of the main metabolite peak (*m/z* 380) was accomplished by MALDI-MS/MS product ion imaging, whereby fragments found in the kidney tissue were directly related to known fragments obtained from the drug standard. PCA confirmed that the drug compound co-localizes with its demethylated and oxidized metabolites.

In summary, we succeeded in demonstrating drug-metabolite correlation directly in a formalin-fixed paraffin-embedded tissue sample by PCA on both MALDI and DESI imagind MS data. Both ionization methods revealed that the abundance of the masses corresponding to the drug compound and its main metabolite was much lower compared with formalin-fixed frozen and non-embedded tissue sections.

## References

[1] McDonnell LA, Heeren RMA (2007). Imaging mass spectrometry. Mass Spectrom. Rev..

[2] Knochenmuss R (2006). Ion formation mechanisms in UV-MALDI. Analyst.

[3] Dreisewerd K (2003). The desorption process in MALDI. Chem. Rev..

[4] Cooks RG, Ouyang Z, Takats Z, Wiseman JM (2006). Detection technologies. ambient mass spectrometry. Science.

[5] Takáts Z, Wiseman JM, Cooks RG (2005). Ambient mass spectrometry using desorption electrospray ionization (DESI): instrumentation, mechanisms, and applications in forensics, chemistry, and biology. J. Mass Spectrom..

[6] Takáts Z, Wiseman JM, Gologan B, Cooks RG (2004). Mass spectrometry sampling under ambient conditions with desorption electrospray ionization. Science.

[7] Goodwin RJA (2012). Sample preparation for mass spectrometry imaging: small mistakes can lead to big consequences. J. Proteomics..

[8] Guerquin-Kern J-L, Wu T-D, Quintana C, Croisy A (2005). Progress in analytical imaging of the cell by dynamic secondary ion mass spectrometry (SIMS) microscopy. Biochim. Biophys. Acta.

[9] Caldwell RL, Caprioli RM (2005). Tissue profiling by mass spectrometry: a review of methodology and applications. Mol. Cell. Proteomics..

[10] Dihazi H, Bohrer R, Jahn O, Lenz C, Majcherczyk A, Schmidt B, Urlaub H, Valerius O, Asif AR (2013). Mass spectrometry imaging: linking molecule profiles to tissue spatial distribution. Expert Rev. Proteomics..

[11] Dill A, Eberlin L, Costa A, Ifa D, Cooks R (2011). Data quality in tissue analysis using desorption electrospray ionization. Anal. Bioanal. Chem..

[12] Lolkema, M.P, Bohets, H.H., Arkenau, H-T, Lampo, A., Barale, E., de Jonge M.J.A., van Doorn, L., Hellemans, P., de Bono, J.S., Eskens, F.A.L.M.: The c-Met tyrosine kinase inhibitor JNJ-38877605 causes renal toxicity through species specific insoluble metabolite formation. Clin. Cancer Res. **21**, 2297–2304 (2015) 10.1158/1078-0432.CCR-14-3258 PMC4433755 25745036

[13] Infante JR, Rugg T, Gordon M, Rooney I, Rosen L, Zeh K, Liu R, Burris HA, Ramanathan RK (2013). Unexpected renal toxicity associated with SGX523, a small molecule inhibitor of MET. Investig. New Drugs.

[14] Nilsson A, Fehniger TE, Gustavsson L, Andersson M, Kenne K, Marko-Varga G, Andrén PE (2010). Fine mapping the spatial distribution and concentration of unlabeled drugs within tissue micro-compartments using imaging mass spectrometry. PLoS One.

[15] Buck, A., Ly, A., Balluff, B., Sun, N., Gorzolka, K., Feuchtinger, A., Janssen, K-P., Kuppen, P.J.K., van de Velde, C.J.H., Weirich, G., Erlmeier, F., Langer, R., Aubele, M., Zitzelsberger, H., Aichler, M., Walch, A.: High-resolution MALDI-FT-ICR MS Imaging for the analysis of metabolites from formalin-fixed paraffin-embedded clinical tissue samples. J. Pathol. **237**, 123–132 (2015) 10.1002/path.4560 25965788

[16] Gorzolka K, Walch A (2014). MALDI mass spectrometry imaging of formalin-fixed paraffin-embedded tissues in clinical research. Histol. Histopathol..

[17] Tyler BJ (2006). Multivariate statistical image processing for molecular specific imaging in organic and bio-systems. Appl. Surf. Sci..

[18] Tyler BJ, Rayal G, Castner DG (2007). Multivariate analysis strategies for processing ToF-SIMS images of biomaterials. Biomaterials.

